# Creating a novel petal regeneration system for function identification of colour gene of grape hyacinth

**DOI:** 10.1186/s13007-021-00794-7

**Published:** 2021-09-16

**Authors:** Qian Lou, Hongli Liu, Wen Luo, Kaili Chen, Yali Liu

**Affiliations:** 1 College of Horticulture, Northwest A & F University, Yangling, 712100 Shaanxi People’s Republic of China; 2 College of Landscape Architecture and Arts, Northwest A & F University, Yangling, 712100 Shaanxi People’s Republic of China; 3 Key Laboratory of Horticultural Plant Biology and Germplasm Innovation in Northwest China, Ministry of Agriculture, Yangling, 712100 Shaanxi People’s Republic of China; 4 State Key Laboratory of Crop Stress Biology in Arid Areas, Northwest A&F University, Yangling, 712100 Shaanxi People’s Republic of China

**Keywords:** *Muscari*, In vitro petal organogenesis, *Agrobacterium* transformation, Gene function analysis

## Abstract

**Background:**

Grape hyacinth (*Muscari* spp*.*) is one of the most important ornamental bulbous plants. However, its lengthy juvenile period and time-consuming transformation approaches under the available protocols impedes the functional characterisation of its genes in flower tissues. In vitro flower organogenesis has long been used to hasten the breeding cycle of plants but has not been exploited for shortening the period of gene transformation and characterisation in flowers.

**Results:**

A petal regeneration system was established for stable transformation and function identification of colour gene in grape hyacinth. By culturing on Murashige and Skoog medium (MS) with 0.45 μM 2,4-dichlorophenoxyacetic acid (2,4-D) and 8.88 μM 6-benzyladenine (6-BA), during the colour-changing period, the flower bud explants gave rise to regeneration petals in less than 3 months, instead of the 3 years required in field-grown plants. By combining this system with *Agrobacterium*-mediated transformation, a glucuronidase reporter gene (*GUS*) was delivered into grape hyacinth petals. Ultimately, 214 transgenic petals were regenerated from 24 resistant explants. PCR and *GUS* quantitative analyses confirmed that these putative transgenic petals have stably overexpressed *GUS* genes. Furthermore, an RNAi vector of the anthocyanidin 3-*O*-glucosyltransferase gene (*MaGT*) was integrated into grape hyacinth petals using the same strategy. Compared with the non-transgenic controls, reduced expression of the *MaGT* occurred in all transgenic petals, which caused pigmentation loss by repressing anthocyanin accumulation.

**Conclusion:**

The *Agrobacterium* transformation method via petal organogenesis of grape hyacinth took only 3–4 months to implement, and was faster and easier to perform than other gene-overexpressing or -silencing techniques that are currently available.

**Supplementary Information:**

The online version contains supplementary material available at 10.1186/s13007-021-00794-7.

## Background

*Muscari*, commonly known as grape hyacinth, is one of the most important ornamental bulbous plants [1]. It is especially popular as a bedding plant and pot plant due to its eye-catching blue colour and musky odour [1, 2]. Currently, the understanding of these flower traits has benefited tremendously from progress made via the field of bioinformatics [3]. There has also been great interest in rapid assay systems to determine gene functions [3–9]. However, the long juvenile period (3–5 years) and the difficulty of producing transgenic plants have restricted the functional analysis of introduced genes in flower tissues [2, 8].

Gene function analysis in flowers remains a challenge for grape hyacinth. For species-specific genes related to flowers, methods using a heterologous model plant system are mainly suitable as ‘forward genetic’ tools and might lead to misleading results. Attempts at transient expression, whether *Agrobacterium* infiltration, gene gun, or virus-induced gene silencing, have failed to achieve stable and repeatable results in grape hyacinth or its closely related species. Although an *Agrobacterium* transformation protocol using somatic embryogenesis has been reported and used to produce transgenic seedlings carrying *GUS* reporter genes [9, 10], no further information is available about the follow-up study on flower traits. Even ignoring the juvenile period, this method takes over 6–8 months to obtain transgenic seedlings [9, 10]. A more effective and rapid transformation protocol in flowers is thus needed.

Organogenesis from transformed explants is the preferred method for the generation of transgenic plants and analysis of gene function in many plant species including grape [11] and peach [12], among others. However, these methods focus on the direct induction of vegetative organs and regeneration of complete transgenic plants. In vitro flower organogenesis is of great potential in shortening the flowering cycle and pre-assessing flower traits, such as colour, shape, and scent. It has long been used to hasten the breeding cycle of plants with long juvenile phases [13] but has not been exploited for gene transfer and rapid function assessment in transgenic flowers.

In this study, an *Agrobacterium*-mediated transformation method was established for petal organogenesis, which enabled the rapid analysis of colour gene function in grape hyacinth.

## Methods

### Plant materials

Five-year-old bulbs of *M. armeniacum* were obtained from Zhejiang Hongyue Seeds Company Limited (Zhejiang, China) and planted in the experimental field at Northwest A&F University (Xi' an, Shaanxi, China).

### Flower organogenesis from explants of grape hyacinth

Grape hyacinth flowers in four different developmental stages were used as explants (Additional file 1a). The flowers excised from the inflorescence were surface sterilised in 75% (v/v) ethanol for 30 s, followed by 0.1% HgCl_2_ for 5 min, and subsequently rinsed twice with sterile water. Sterilised explants were then cultured on petal induction media consisting of Murashige and Skoog medium (MS) [14] supplemented with 3% (w/v) sucrose, 0.3% (w/v) phytagel, 0.45 μM 2,4-dichlorophenoxyacetic acid (2,4-D), and different concentrations of 6-benzyladenine (6-BA; 0.00, 1.11, 2.22, 4.44, 8.88, 13.32, 17.76 μM), and subcultured every 2 weeks. The media were sterilised at 121 °C for 20 min, and the pH was adjusted to 6.0. All cultures were grown at 21 °C under a 10/14 h light/dark photoperiod with a light intensity of 25 μmol m^−2^ s^−1^. The number of regenerated petals was recorded after 11 weeks to compare the efficiency of petal organogenesis.

### *Agrobacterium* strain and plant expression vectors

pFGC5941 carrying *GUS* regulated by the CaMV 35S promoter was generated as previously described [15]. For the *MaGT1* RNAi expression vector, the 405 bp partial coding region of *MaGT1* (GenBank No. MK652470) was amplified using the specific primers RNAi-F and RNAi-R (Additional file 2). *Nco*I and *Pac*I restriction enzyme sites and protective bases were added to the 5′ end of the forward primer RNAi-F. At the same time, *Asc*I and *Xba*lI restriction enzyme sites and protective bases were added to the 3′ end of the reverse primer RNAi-R. The digested *MaGT1* fragments were inserted into the *Asc*I/*Nco*I and *Pac*I/*Xba*lI enzyme sites of the vector pFGC5941 at inverted repeat sequences to form a plant RNAi expression vector, pFGC-*MaGT1 RNAi*, which can generate a hairpin RNAi construct. Then, all vectors were introduced into *A. tumefaciens* strain LBA4404.

Each bacterial strain was grown in yeast extract-peptone (YEP) liquid medium supplemented with 60 μg mL^−1^ rifampicin, 50 μg ml^−1^ streptomycin, and 50 μg mL^−1^ kanamycin at 28 °C for 2 days. The cultures were then grown in 50 mL of YEP at 28 °C overnight. Bacterial cells were harvested after centrifugation at 5500 rpm for 5 min and then resuspended to an OD_600_ of 0.6 in infiltration solution, which consisted of 1/2 MS medium supplemented with 150 μM acetosyringone for infiltration into the grape hyacinth explants.

### *Agrobacterium* transformation

After 5 days of pre-culture in co-cultivation medium [MS medium containing 0.45 μM 2,4-D, 8.88 μM 6-BA, 3% (w/v) sucrose, 0.3% (w/v) phytagel], the surface-sterilised flower buds at stage I (Additional file 1a) were submerged in 50 mL centrifuge tubes containing 20 mL of the infiltration solution, and then ultrasonicated (Ultraviolet–visible spectrophotometer, Shanghai, China) at 80 MHz for 5 min. The sonicated explants were incubated with the *A. tumefaciens* suspension harbouring the target plasmid (OD_600_ = 0.6) for 5 min. The redundant bacterial liquid was removed from the surface of the flower buds with sterile paper. Then, the dried flower buds were cultured on co-cultivation media for 3 days at 24 °C in the dark. Subsequently, the infected explants were rinsed in liquid 1/2 MS containing 500 μg mL^−1^ cefotaxime for 10 min to remove the overgrown *Agrobacterium* and transferred onto MS medium containing 0.45 μM 2,4-D, 8.88 μM 6-BA, 3% (w/v) sucrose, 0.3% (w/v) phytagel, and 500 μg mL^−1^ cefotaxime for 7-day delayed selection. The cultures were transferred onto a similar medium supplemented with 0.5 mg L^−1^ bialaphos (BIA) (Meiji Seika, Tokyo, Japan) for the selection of transformed tissues. These were sub-cultured every 2 weeks onto fresh selection medium. The cultures were grown at 21 °C under a 10/14 h light/dark photoperiod. After 11–15 weeks of screening cultures, the resistant petals that developed from the explants were picked out prior to full expansion (about ½–2/3 of their full size, fully pigmented), and used for further analysis.

### Molecular analysis of transgenic plants

Prior to full expansion, the non-transgenic and putative transgenic petals were randomly excised for molecular analysis. Genomic DNA was extracted from 100 mg of each petal sample using a TIANamp Genomic DNA kit (TIANGEN Biotech Co., Ltd., Beijing, China) following the manufacturer’s instructions.

PCR was carried out to detect the target sequences (the *GUS* or Basta gene fragment) in transgenic petals with the specific primers (Additional file 2) according to the rTaq manufacturer’s instructions (Takara Biotechnology, Dalian, China). The plasmid DNA used in transformed plants served as a positive control, while DNA from non-transgenic petals served as a negative control.

Gene expression analysis was conducted by semi-quantitative RT-PCR and real-time RT-PCR. For each sample, total RNA extraction, cDNA synthesis, and a qRT-PCR assay were undertaken using the protocols described by Liu et al. [7]. Semi-quantitative RT-PCR was conducted using the following parameters: 94 °C for 3 min; 25 cycles at 94 °C for 30 s, 55 °C for 30 s, and 72 °C for 30 s; followed by final elongation at 72 °C for 5 min. The primers for qRT-PCR and the internal control genes, *MaActin*, are listed in Additional file 2. Analysis was performed on at least three biological replicates.

### GUS assay

*GUS* histochemical location was conducted as previously described [1]. Each photograph in Fig. 2f is representative of at least nine tissues from three replicated experiments. Fluorometric quantitative analysis was measured according to Jefferson’s method [16]. The control or transgenic samples were analysed to determine GUS activities with the substrate of 1 mM 4-methyl umbelliferyl *β*-d-glucuronide (Sigma-Aldrich, Shanghai, China). Fluorescence values were recorded with a Hitachi 850 Fluorescence spectrophotometer (Hitachi, Tokyo, Japan). The protein concentration was determined as described by Bradford [17].

### Morphological observations

The cross-sections and protoplasts of regenerated and field-grown petals were made by hand sectioning and the enzymolysis method, respectively, as previously described [18]. For preparation of free protoplasts, tepal strips (0.5 mm) were cut from the fully pigmented petals and quickly immersed in enzyme solution containing 1.3% (w/v) cellulase R-10 (Yakult, Japan), 0.3% (w/v) macerozyme R-10 (Yakult, Japan), 0.8 M mannitol, 80 mM KCl, and 20 mM MES-Tris (pH 6.0). After vacuum infiltration for 30 min, digestion was continued in the dark for 3 h at room temperature. Then the released protoplasts were further purificated from the reaction mixture by filter and centrifugal separation/re-suspension. The fresh cross-section tissues and protoplasts were immediately examined using a light microscope (Eclipse 50i, Nikon, Japan).

### Anthocyanin analysis

The method for anthocyanin extraction and quantification was conducted as previously described [1]. In brief, the anthocyanins were extracted from the transgenic and non-transgenic petals using a methanol:water:formic acid:TFA solution (70:27:2:1, v/v). The extracts were treated with an equal volume of 6 M HCl at 90 °C to yield anthocyanin hydrolysates. Then, high-performance liquid chromatography (HPLC) was performed following the previously described method [1]. The anthocyanin content was determined using delphinidin equivalents (Sigma, St. Louis, Mo, USA). All samples were analysed in three biological replicates.

### Statistical analyses

The means and error bars (SD) were obtained from at least three independent experiments. Significant differences were assessed by one-way analysis of variance (ANOVA) and the Student’s t-test using SPSS 20.0 software (SPSS Inc., Chicago, USA).

## Results

### Explant age and cytokinin is critical for in vitro petal organogenesis

In general, the ratio between exogenous auxin and cytokinin determines the type of regenerated organ [19]. Therefore, the flower buds were cultured on six petal induction media containing 0.45 μM 2,4-D and different concentrations of 6-BA (0–17.76 μM). Without 6-BA, no organs regenerated from the explants on the medium containing only 2,4-D after 11 weeks of culture (Additional file 1b, Table 1). Supplementation with 6-BA evidently enhanced petal organ formation. When the ratio of 6-BA:2,4-D rose from 2.5 to 19.7, the petal organogenesis efficiency increased from 0 to 36.8% (Additional file 1b, Table 1). However, with further increasing concentrations of 6-BA, the regenerated organs were progressively aberrant, becoming thickened and deformed (Additional file 1b, Table 1). Therefore, in subsequent experiments, 8.88 μM 6-BA was chosen to investigate the influence of explant age on the efficiency of petal organogenesis.

**Table 1:** Effect of exogenous 6-BA on flower petal regeneration of grape hyacinth

6-BA concentration (μM)	Frequency of petal regeneration (%)	Average petal number per explant
0.00	0.0 ± 0.0^c^	0.0 ± 0.0^b^
1.11	0.0 ± 0.0^c^	0.0 ± 0.0^b^
2.22	0.0 ± 0.0^c^	0.0 ± 0.0^b^
4.44	3.1 ± 2.0^c^	5.0 ± 1.0^b^
8.88	36.8 ± 4.7^a^	11.7 ± 2.1^a^
13.32	13.9 ± 4.3^b^*	4.3 ± 1.5^b^*
17.76	0.8 ± 0.8^c^*	0.3 ± 0.6^c^*

^a^Calculated on the 11th week after stage a flower explants were cultured on MS medium containing 0.45 μM 2,4-D and different concentrations of 6-BA

^b^Means ± SD within a column followed by the same letter are not significantly different according to Tukey’s multiple range test at P < 0.05

^c^*Thicker, deformed, and slow-growing organs (Fig. 1i)

The explants excised from reproductive organs seemed to be vital for flower neoformation [20]. The previous research showed that petals of grape hyacinth could be induced from the flower bud explants rather than from any vegetative explants (data not shown). Therefore, flower buds of different developmental stages were cultured on media with 0.45 μM 2,4-D and 8.88 μM 6-BA. Flower development was divided into four stages (Additional file 1a): stage I, white, closed flower buds; stage II, flower buds during the colour-changing period; stage III, fully pigmented closed buds; stage IV, completely opened flowers. Explants of stage I gave rise only to leaves, whereas explants of stages II and III successfully produced petals and/or deformed petal-like structures (Additional file 1a, Table 2). The highest frequency (57%) of petal formation was obtained with flower buds of stage II (Table 2). Most of the stage IV explants developed into swollen fruits or gradually became brown and died during culture (Additional file 1a).

**Table 2:** Frequency of flower petal regeneration from explants of different ages

Flower stage	Frequency of flower petal regeneration (%)	Average petal number per explant
a	0.0 ± 0.0^c^	0.0 ± 0.0^b^
b	57.0 ± 11.0^a^	10.7 ± 4.0^a^
c	19.7 ± 10.1^b^	9.0 ± 6.2^a^
d	0.0 ± 0.0^c^	0.0 ± 0.0^b^

^a^Calculated on the 11th week after the explants were cultured on MS medium containing 0.45 μM 2,4-D and 8.88 μM 6-BA

^b^Means ± SD within a column followed by the same letter are not significantly different according to Tukey’s multiple range test at P < 0.05

Thus, the flower explants of stage II and petal induction media containing 0.45 μM 2,4-D and 8.88 μM 6-BA were used in later genetic transformation studies.

### In vitro petal regeneration of grape hyacinth

**Fig. 1:**
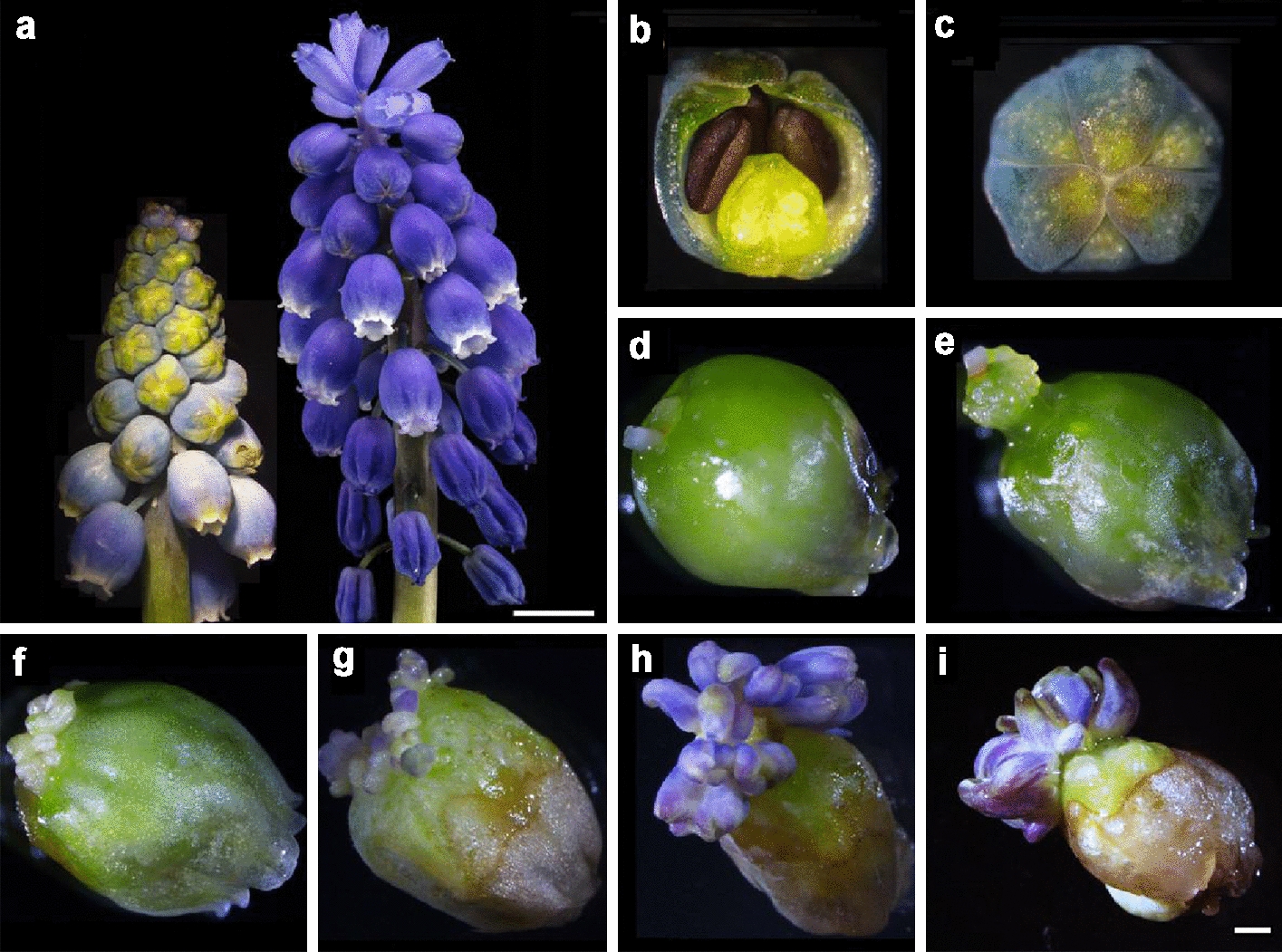
In vitro petal regeneration of grape hyacinth. Young and mature inflorescence of grape hyacinth (**a**), flower bud during the colour-turning period of flower organ induction (**b**, **c**), the explants swelled and turned green (**d**), callus formation (**e**), flower petal primordia formation (**f**), a break in the colour of regenerated petals (**g**), fully pigmented regenerated petals (**h**), petal-like structures (**i**). Scale bar in a: 0.5 cm, Scale bar in **b**–**i**: 100 μm

Within 5 days, the flower explants of stage II swelled and turned green in cultures on media containing 0.45 μM 2,4-D and 8.88 μM 6-BA (Fig. 1c, d). Dedifferentiation subsequently took place, consisting of rapid cell division in several cells located around the flower stalk and the production of an aggregate of meristematic cells (Fig. 1e). After this, meristematic cells gave rise to petal primordia from which visible petals appeared 5 weeks from the start of culture (Fig. 1f). The formation of petal primordia continued in transfers to the same medium, resulting in the emergence of multiple petals (Fig. 1g). After 6 weeks of culture, these petals gradually expanded and turned violet-blue from the top surface, finally developing into fully pigmented, rolling inward petals or thicker petal-like structures (Fig. 1h, i). The regenerated petals were small in size but similar in structure to the field-grown petals. They both had an outer epidermis layer, a spongy layer and a palisade layer that consists of pigment cells (Additional file 3). Different shades of purple and blue protoplasts were superimposed giving rise to the same violet-blue petal colours (Additional file 3).

### Generation of grape hyacinth petals overexpressing the GUS gene

**Fig. 2:**
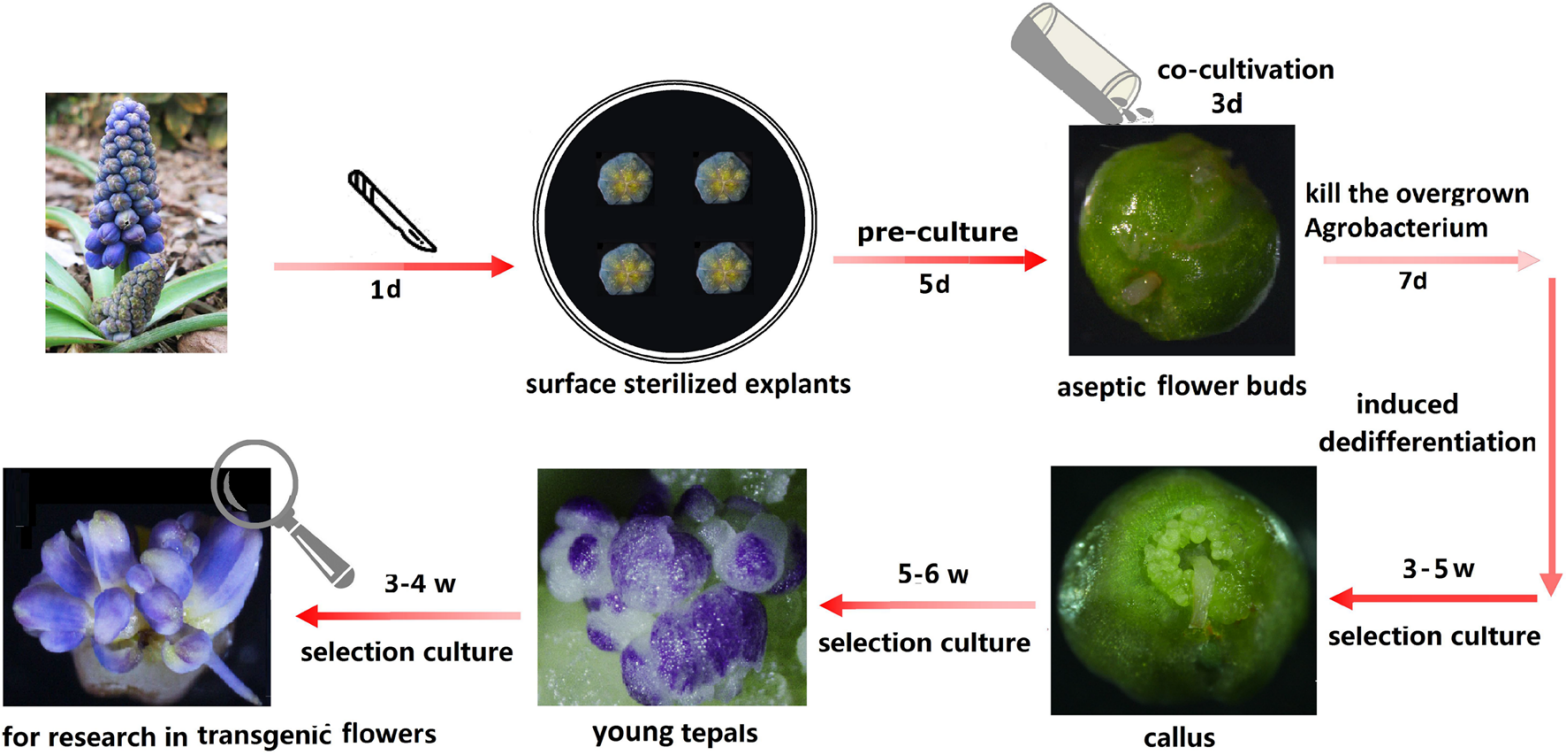
Flowchart of *Agrobacterium* mediated transformation methods via petal organogenesis system

**Fig. 3:**
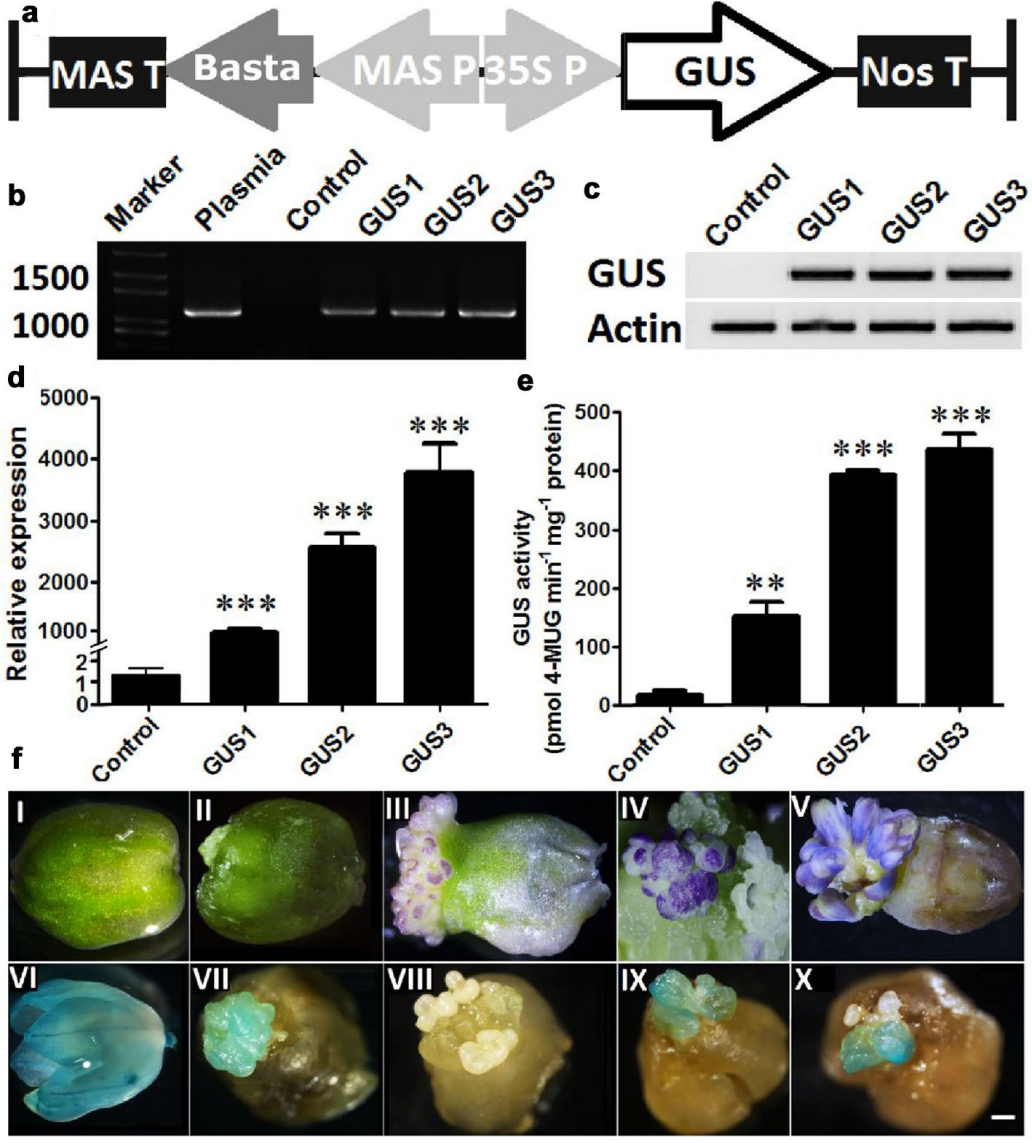
Overexpression of the *GUS* gene in transgenic petals of grape hyacinth. The *GUS* gene was ligated into the vector pFGC5941 under the control of the CaMV 35S promoter. The *Basta* gene was used as a selection marker gene (**a**). PCR amplification of a 1216 bp DNA fragment of the *GUS* gene from the transgenic and non-transgenic lines (**b**). Total RNA was extracted from young petals prior to full expansion. Then, the mRNA accumulation of the *GUS* gene was assessed by semi-quantitative and quantitative real-time PCR. *Actin* was used as a reference gene (**c**, **d**). GUS protein activity in the transgenic and non-transgenic petals (**e**). Each bar represents means ± standard deviation from three dependent replicates. The symbols ‘**’ and ‘***’ above bars indicate statistically significant differences at P ≤ 0.01 and P ≤ 0.001, respectively, by the student’ s t-test. Control: non-transgenic petals, GUS1-3: transgenic petals overexpressing the *GUS* gene. Production of transgenic grape hyacinth petals via *Agrobacterium* mediated transformation and flower organogenesis system (**f**). After pre-culture and co-cultivation with *A. tumefaciens* carrying CaMV35S::GUS, the flower explants turned green (**I**). The majority of them showed transient GUS expression (**VI**). Then, the BIA^r^cell clusters developed around the flower stalk on the selection medium (**II**). Histochemical staining indicated that these cell clusters showed GUS activity (**VII**). BIA^r^ cell cluster gave rise to violet-blue petals (**III, IV**, **V**). The non-transgenic and BIA^r^ petals, prior to full expansion, were excised and used for later analysis (**IV**). The GUS histochemical assay showed that blue staining was detected in resistant petals (**IX**, **X**), whereas no blue spots were observed in the non-co-cultivated controls (**VIII**). Scale bar in d: 100 μm

By combining the petal regeneration system with the *Agrobacterium*-mediated transformation method (Fig. 2), a glucuronidase reporter gene (*GUS*) was delivered into grape hyacinth. First, the flower buds of stage II (Additional file 1a) were co-cultivated with *A. tumefaciens* carrying CaMV35S::GUS for 3 days (Fig. 3a). To determine the efficiency of transient transformation, the GUS assay was carried out on 60 explants after co-cultivation, of which 92% showed a high level of blue staining (Fig. 3f VI), whereas few or no blue spots were observed in the non-co-cultivated controls. Meanwhile, the other *Agrobacterium*-infected explants were transferred onto a medium containing cefotaxime for 7 days to kill the overgrown bacteria. Then, they were successively sub-cultured onto a selection medium containing 0.5 mg L^−1^ BIA. Three to 5 weeks later, BIA^r^ cell clusters started to develop around the flower stalk (Fig. 3f II). Histochemical staining showed that 26 of 30 BIA^r^ clusters had *GUS* expression activity (Fig. 3f VII). In the following 5 to 6 weeks, the majority resistant calli differentiated into numerous petals (Fig. 3f III), which expanded and turned violet-blue within 3 to 4 weeks (Fig. 3f IV, V). Overall, 24 of 200 explants produced BIA^r^ petals, thus yielding a transformation efficiency of 12%. On average, 8.9 resistant petals were regenerated from each explant. No apparent phenotypic alterations were observed between resistant and non-transgenic petals. All detected BIA^r^ petals showed GUS staining (Fig. 3f IX, X), whereas no GUS expression could be found in those petals from non-transgenic samples (Fig. 3f VIII). Similarly, semi-quantitative and quantitative real-time PCR showed that the GUS activities of BIA^r^ petals were significantly higher than those of the non-transformed control (Fig. 3c, d). The relative mRNA expression levels of the *GUS* gene in the transformants were over 1000-fold greater than those in the control (Fig. 3d). PCR analysis confirmed that the expected *GUS* bands of 1216 bp were present in the recombinant DNA of putative transgenic petals (Fig. 3b).

### Silencing *MaGT* reduced anthocyanin accumulation in regenerated petals of grape hyacinth

**Fig. 4:**
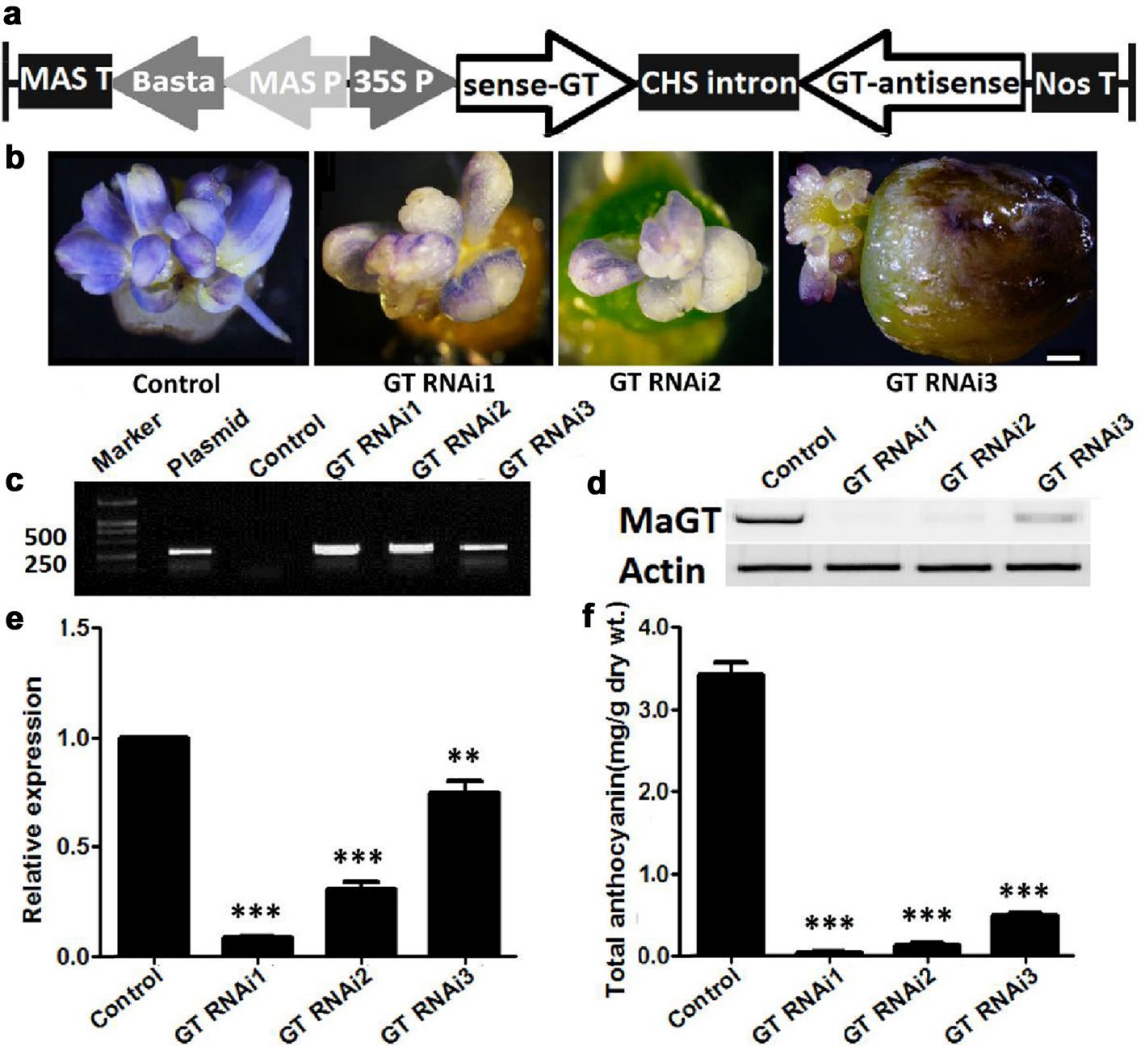
Silence of the *MaGT* gene in transgenic petals of grape hyacinth. Schematic representation of RNAi expression vectors, pFGC-*MaGT1 RNAi*, constructed for transformation (**a**). Silencing of *MaGT* in transgenic flower petals resulted in a clear phenotypic change in coloration (**b**). Shown are non-transformed controls (Control) and three *MaGT* gene-silenced lines. Scale bar in d: 100 μm. PCR amplification of a 281 bp DNA fragment of the *Basta* gene from the transgenic and non-transgenic lines (**c**). Total RNA was extracted from young petals prior to full expansion. Then, mRNA accumulation of the *MaGT* gene was assessed by semi-quantitative and quantitative real-time PCR. *Actin* was used as a reference gene (**d, e**). Anthocyanin analysis of non-transformed and transformed flower petals (**f**). Each bar represents means ± standard deviations from three dependent replicates. The symbols ‘**’ and ‘***’ above bars indicate statistically significant differences at P ≤ 0.01 and P ≤ 0.001, respectively, by the student’s t-test. Control: non-transgenic petals, GT RNAi1-3: transgenic petals silencing *GUS* gene expression

The anthocyanidin 3-*O*-glucosyltransferase gene (*MaGT*, gene bank accession No. MK652470)*,* a crucial gene in flower pigmentation, was cloned to construct the RNAi vector (Fig. 4a) and transformed into petals as described previously. In brief, after co-cultivation and bacteria-elimination, the flower buds were transferred onto selection medium to produce BIA^r^ calli. Then, these calli formed numerous resistant petals. Moreover, 187 BIA^r^ petals were obtained from 34 explants within the following 8–10 weeks. Compared with the non-transgenic controls (violet-blue), all BIA^r^ petals showed less pigmentation (pale purple, Fig. 4f). PCR analysis showed that the expected Basta bands of 281 bp were detected in the DNA of all detected samples (Fig. 4c). Significantly reduced expression of *MaGT1* in these transgenic samples compared with that in the control was confirmed by semi-quantitative and quantitative real-time PCR (Fig. 4d, e). Moreover, HPLC also showed that the anthocyanin content decreased significantly in transformed petals (Fig. 4f). The results indicated that silencing of *MaGT* effectively decreased flower petal pigmentation by reducing anthocyanin accumulation in grape hyacinth.

## Discussion

### A petal regeneration system in grape hyacinth

Grape hyacinths require over 3–5 years of vegetative growth before flowering [2, 8], which restricts the efficiency of functional analysis for introduced genes. In this study, a petal regeneration method was developed to significantly shorten the time. Under optimal conditions, flower petals could be observed less than 3 months after in vitro explants were cultured, instead of the 3 years required in field-grown plants.

Early in 1957, Skoog and Miller revealed that the ratio between auxin and cytokinin determines the nature of regenerated organs during in vitro culture [21]. In grape hyacinth, when the flower buds were cultured on a medium with 0.45 μM 2,4-D, increasing the 6-BA concentration evidently enhanced petal neoformation. However, when the explants were transferred to a medium with a lower 6-BA and/or 2,4-D concentration, the formation of petal primordia was replaced by that of leaves and callus (data not shown). Similar phenomena were observed in *Hyacinthus orientalis* [22]. At reduced levels of both cytokinin and auxin, the formation of regenerated petals ceased and was replaced by that of stamens and/or carpels [22]. For *Oncidium* ‘Sweet Sugar’, the decrease in the ratio of Thidiazuron (TDZ) and NAA retarded proliferation of yellowish petal-like structures from flower-stalk callus [23]. These results demonstrated that a high cytokinin:auxin ratio at a appropriate concentration can act as a trigger for petal neoformation. In contrast to *Oncidium* ‘Gower Ramsey’, over-supplementation of dicamba, an auxin, significantly enhanced the induction of abnormal embryos, which developed into petals rather than scale leaves [24]. In view of the fact that petals are just morphological modified leaves, the difference might be related to the effect of dicamba on morphological abnormality on somatic embryogenesis [24], which need further research.

Another vital factor for in vitro flower organogenesis is explant type. A number of studies have pointed out that the use of explants excised from reproductive organs might be an important condition for in vitro flower organ regeneration [20, 22, 23, 25, 26]. In *Dracaena fragrans*, the flower segments directly regenerate petal, flower bud, inflorescence, inflorescence branch, and leaf tissues under culture conditions, whereas the segments of vegetative organs can only regenerate vegetative tissues [25]. For *Hyacinthus orientalis*, in the relatively early and later developmental stages, perianth explants cultured on the same medium tend to differentiate into petals and stamens/ovules, respectively [26]. In this research, neither bulb, scales, leaves, peduncles, nor perianths could regenerate flower organs (data not shown). Only the young flower buds were suitable for petal regeneration, which reconfirmed the importance of explant nature and age in flower organ neoformation.

### An *Agrobacterium*-mediated transformation method suitable for the rapid assessment of gene function in flowers

Combined with the petal regeneration system and *Agrobacterium*-mediated transformation, a useful method was established to accelerate the analysis of colour gene function in grape hyacinth petals. This method can be used to overexpress *GUS* reporter genes and silence the functional gene *MaGT* in petals. GUS staining and novel colours were observed in these transgenic petals, hence the early assessment of petal colour, pigment components, and gene expression is possible. By using this method, a specific gene can be overexpressed or silenced in grape hyacinth petals within 3–4 months (Fig. 2), and the transgenic petals can be maintained for 1 month for use in various analyses. In addition to these advantages, petal regeneration from explants skips the juvenile stages. This will, in turn, reduce labour costs and optimise the space required for precious transformation methods [9, 10]. Taken together, these abilities make the protocols described here a pioneering method for both forward and reverse screening for gene function analyses in grape hyacinth flowers. To our knowledge, this is the first reported case of an in vitro method to rapidly determine gene function that combines the advantages of *Agrobacterium*-mediated transformation and petal regeneration systems, giving the method great potential. Unlike embryogenesis, flower morphogenesis from organ explants has been developed in more than 40 families, 75 genera, and over 100 species [27]. In fact, early in 1973, Tran Than Van observed in vitro flower regeneration from the thin cell layers of *Nicotiana tabacum* [20]. In the following century, almost all flower organs were successively obtained by in vitro organogenesis, including inflorescences [28, 29], entire flowers [20, 30], petals [26, 31], stamens [26], pistils [32], stigmas [33], carpels [34], and ovules [26], among others. These established flower organogenesis systems may be easily adapted into genetic transformation protocols and used to hasten the gene characterisation cycle in other flower species.

The method described here also has limitations. First, this technique is limited in the range of species and tissues. Most notably, stable transformation via petal morphogenesis cannot be used to study the target gene function in other organs, such as leaves or roots. Furthermore, the successful spread and application of this method requires diverse regeneration systems tailored to specific needs. Second, continuous and high 6-BA concentrations may cause the formation of abnormal petals (Fig. 1h). In addition, if the study focuses on the cytokinin signaling pathway, it is advisable to choose an alternative method for functional analysis. Third, the propagation coefficient of transgenic petals was relatively low. Thus, unless an obvious change in phenotype is established, it is difficult to obtain enough transformed cells to enable various assays on the impact of the transgene. Fourth, explant collection and transformation work must be arranged in a finite time horizon because flowering in grape hyacinth only occurs for a few weeks in spring [8]. Fifth, when organogenesis rather than embryogenesis is used to regenerate transformants from explants co-cultured with *Agrobacteria*, the risk of regenerating chimeral or non-true-to-type transgenic tissues increase. Sixth, there were some nonuniform characteristics of transformed petals caused by transgene location effect and copy number. Therefore, a number of replicates is needed to obtain meaningful results.

## Conclusions

In conclusion, the novel system for obtaining regenerated petal of grape hyacinth was set up through this study, which was beneficial to the stable transformation and identification of colour gene in petals. Besides, it is faster and easier to perform than other gene-overexpressing or -silencing protocols that are currently available.

## Supplementary Information

**Additional file 1. **Effect of explant age and 6-BA concentration on flower petal regeneration of grape hyacinth.

**Additional file 2. **List of primers used in this study.

**Additional file 3. **Cellular features of grape hyacinth petals in vitro and in vivo.

## Data Availability

The authors are pleased to share analysed/raw data and plant materials upon reasonable request.
