# Supplementary material for: Creating a novel petal regeneration system for function identification of colour gene of grape hyacinth
Source: Plant Methods. 2021 Sep 16;17:94. doi: 10.1186/s13007-021-00794-7 (PMC8444494; doi:10.1186/s13007-021-00794-7)
Supplement: Supplementary file 1 — Additional file 1. Effect of explant age and 6-BA concentration on flower petal regeneration of grape hyacinth. [file 13007_2021_794_MOESM1_ESM.docx]

**Additional Material**


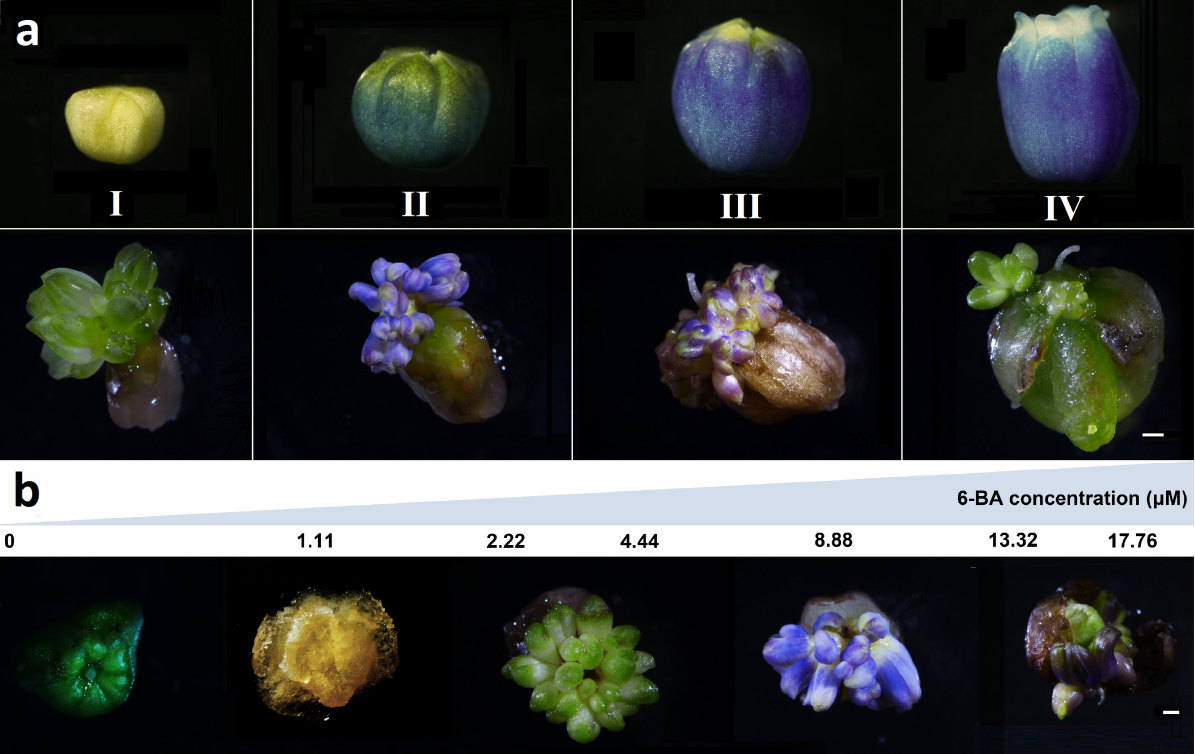


**Additional file 1** Effect of explant age and 6-BA concentration on flower petal regeneration of grape hyacinth. (**a**) In vitro organogenesis from four different flower explants of grape hyacinth. After culture on MS medium containing 0.45 μM 2,4-D and 8.88 μM 6-BA for 11 weeks, white, closed flower buds regenerated leaves (**I**). Flower buds during the turning period regenerated petals (**II**). Fully pigmentation buds regenerated aberrant petal-like structures (**III**). Completely opened flowers regenerated leaves (**IV**). (**b**) Effect of different concentrations of 6-BA on the type of regenerated organs. The flower bud explants were cultured on MS medium containing 0.45 μM 2,4-D and different concentrations of 6-BA for 11 weeks. With the gradual increase of 6-BA concentration from 0.00 μM to 17.76 μM, the types of regenerated organs changed in the following order: nothing, calli, vegetative buds, flower petals, aberrant organs. Scale bar: 100 μm
