# Supplementary material for: Creating a novel petal regeneration system for function identification of colour gene of grape hyacinth
Source: Plant Methods. 2021 Sep 16;17:94. doi: 10.1186/s13007-021-00794-7 (PMC8444494; doi:10.1186/s13007-021-00794-7)
Supplement: Supplementary file 2 — Additional file 2. List of primers used in this study. [file 13007_2021_794_MOESM2_ESM.docx]

**Additional material**

**Additional file 2** List of primers used in this study.

| **Primer name** | **Primer sequence (5'→3')** |
| --- | --- |
| *MaGT RNAi-F1* | GGGGCCATGGGCTGCCGTTCCTGTGGACTCTGA |
| *MaGT RNAi-R1* | GGCGCGCCGCCTCTCTCGCTTTGTCCCTCA |
| *MaGT RNAi-F2* | GGTTAATTAACTGCCGTTCCTGTGGACTCTGA |
| *MaGT RNAi-R2* | GCTCTAGAGCCTCTCTCGCTTTGTCCCTCA |
| *Basta-PCR-F* | CCCGATGACAGCGACCA |
| *Basta-PCR-R* | TCAACTTCCGTACCGAGCC |
| *GUS-PCR-F* | AACGGCAAGAAAAAGCA |
| *GUS-PCR-R* | GCGAGGTCGCAAAATC |
| *GUS-qRT-F* | GCGTGGTGATGTGGAGTATT |
| *GUS-qRT-R* | TGGTATCGGTGTGAGCGT |
| *MaGT-qRT-F* | CCACCAAAGATGATGGCTACT |
| *MaGT-qRT-R* | GCTTGTACTCGCTCCCAAA |
| *MaActin-qRT-F* | AACATTCAGAAAGAGTCCACCC |
| *MaActin-qRT-R* | GCTTACCAGCAAAGATCAACCG |
