# Supplementary material for: Creating a novel petal regeneration system for function identification of colour gene of grape hyacinth
Source: Plant Methods. 2021 Sep 16;17:94. doi: 10.1186/s13007-021-00794-7 (PMC8444494; doi:10.1186/s13007-021-00794-7)
Supplement: Supplementary file 3 — Additional file 3. Cellular features of grape hyacinth petals in vitro and in vivo. [file 13007_2021_794_MOESM3_ESM.docx]

**Additional material**

**
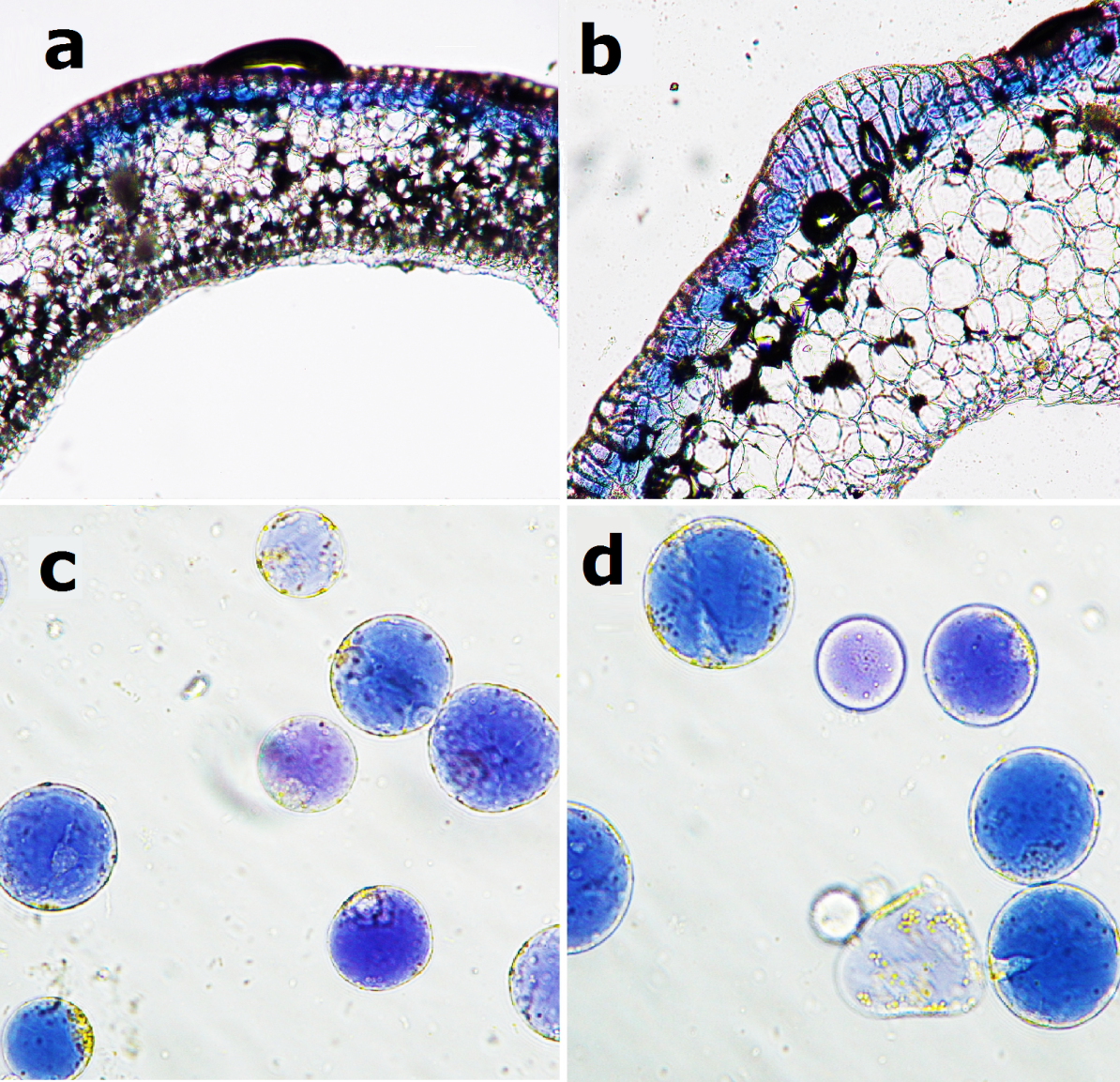
**

**Additional file 3** Cellular features of grape hyacinth petals in vitro and in vivo. Cross sections of in vitro and in vivo tepals (**a**, **b**). Protoplasts from in vitro and in vivo tepals (**c**, **d**)
